# Environmental factors modulated ancient mitochondrial DNA variability and the prevalence of rheumatic diseases in the Basque Country

**DOI:** 10.1038/s41598-019-56921-x

**Published:** 2019-12-31

**Authors:** I. M. Laza, M. Hervella, M. Neira Zubieta, C. de-la-Rúa

**Affiliations:** 1 Department of Genetics, Physical Anthropology and Animal Physiology, Faculty of Science and Technology, University of the Basque Country (UPV-EHU), 48940 Leioa, Bizkaia, Basque Country, Spain; 2 QarK Arqueología, 01006 Vitoria-Gasteiz, Álava, Basque Country, Spain

**Keywords:** Biological anthropology, Population genetics, Genetics, Palaeoclimate

## Abstract

Among the factors that would explain the distribution of mitochondrial lineages in Europe, climate and diseases may have played an important role. A possible explanation lies in the nature of the mitochondrion, in which the energy generation process produces reactive oxygen species that may influence the development of different diseases. The present study is focused on the medieval necropolis of San Miguel de Ereñozar (13^th^–16^th^ centuries, Basque Country), whose inhabitants presented a high prevalence of rheumatic diseases and lived during the Little Ice Age (LIA). Our results indicate a close relationship between rheumatic diseases and mitochondrial haplogroup H, and specifically between spondyloarthropathies and sub-haplogroup H2. One possible explanation may be the climate change that took place in the LIA that favoured those haplogroups that were more energy-efficient, such as haplogroup H, to endure lower temperatures and food shortage. However, it had a biological trade-off: the increased risk of developing rheumatic diseases.

## Introduction

Various pieces of evidence suggest that mitochondrial dysfunction could influence the pathogeny of some human diseases, including neurodegenerative disorders^1^, metabolic diseases^2^, rheumatic pathologies^3,4^, processes associated to age^5^ and cancer^6^. A possible explanation lies in the functionality of the mitochondrion, which is a cellular organelle responsible for supplying most of the energy required for cellular activity, turning nutritional molecules into ATP through oxidative phosphorylation^7^. Mitochondrial metabolism produces Reactive Oxygen Species (ROS) and reactive metabolic intermediates, which are signals that transmit information between the mitochondrion and the nucleus^8,9^, and they can modify the expression of nuclear genes, altering numerous cellular processes and metabolic routes that may influence the development of different diseases^10^.

The present study is focused on the analysis of rheumatic diseases and the role of mitochondrial lineages in their development and prevalence, given the associations provided by previous works^3,4^. It has been observed that mitochondrial function is altered in chondrocytes of individuals with osteoarthritis (OA)^3,4^, producing oxidative stress and increasing chondrocyte apoptosis and cartilage degradation^4,11,12^.

To date, some studies have analysed the relationship between mitochondrial haplogroups and rheumatic diseases. It has been stated that haplogroups H and U are significantly related to a higher risk of developing degenerative bone diseases and with greater severity^13–18^, whereas haplogroups J and T are significantly related to a decrease in the incidence and progression of OA^13–15,17,19–21^, with haplogroup J being also associated with psoriatic arthritis (PsA)^22^. However, no significant association has been found between European mitochondrial haplogroups and rheumatoid arthritis (RA)^18^.

The role of haplogroup H in the pathogenesis of rheumatic diseases is due to the fact that, as it shows a high energy efficiency, it generates a greater oxidative stress, increasing the production of ROS, which causes the degradation of the cartilage and the risk of developing degenerative bone diseases^13–18^. On the other hand, the possible protective effect described for haplogroups T and J lies in a lower energy efficiency in the process of converting calories into ATP, which generates a lower production of ROS and a lower oxidative damage. This mechanism protects the cell from apoptosis and decreases the degeneration of the cartilage involved in the pathogenesis of OA^23,24^. It has been suggested that the differential association of some mitochondrial haplogroups with rheumatic pathologies could be related to an adaptation process of *Homo sapiens* to cold climates, when this species migrated out of Africa^25–27^. Haplogroup H is currently the most common and diverse mitochondrial lineage in Europe (55–40%), which originated 25,000–30,000 years ago in Southwest Asia and entered Europe from the Near East before the Last Glacial Maximum (LGM) (~22,000 years)^28–32^. Haplogroup U, present in 11% of current Europeans, is the oldest maternal lineage in Europe and emerged in Southwest Asia around 55,000 years ago from where it expanded to Europe^28,32–34^. Both haplogroups subsisted in the glacial refugia of Southwestern Europe during the LGM, after which they re-expanded, thus their frequencies were molded by different demographic and cultural factors. Some mitochondrial variants, which were critical for human adaptation in different environments, could have favoured the survival and reproduction of populations that lived in particular climate areas, whereas in other regions they may have not been adaptative^35^.

Given the high prevalence of rheumatic diseases found in the medieval necropolis of San Miguel de Ereñozar (Ereño, Bizkaia, Spain, 13^th^–16^th^ centuries), in the present study we analyse the possible relationship between these diseases and the different mitochondrial lineages, considering the influence of the Earth’s temperature decrease during the Little Ice Age (14^th^–19^th^ centuries). During this period, there was a marked temperature decrease in the Northern Hemisphere, which had negative consequences for survival^36^, since the European glaciers advanced in the mountain valleys and the rainfall and floods increased, which caused bad harvests, famine, conflict, epidemics and increased mortality^37–40^. We believe that these conditions could have influenced the energy production process of mitochondria, promoting mitochondrial dysfunction and the development of different rheumatic diseases.

## Materials

In the present study, we analysed the human bone remains of 163 individuals recovered from the medieval site of San Miguel de Ereñozar (13^th^–16^th^ centuries). This necropolis is located in the northeast of the province of Bizkaia (Basque Country, Spain), in the region of Busturialdea-Urdaibai, which is composed of small urban nuclei; the studied necropolis is in one of these urban nuclei.

A total of 47 out of 163 individuals recovered from the necropolis presented rheumatic manifestations. For comparative purposes, another control group of 43 individuals was selected according to the following criteria: (1) adult (>45 years), (2) absence of rheumatic bone manifestations, and (3) well-preserved remains to allow the molecular analysis.

Since this study is focused on rheumatic pathologies, two differentiated groups were established: (1) individuals without pathological bone manifestations (N = 43), and (2) individuals who showed pathological joint manifestations (N = 47), with diagnoses of entities such as SpA, OA and RA; in few cases it was not possible to reach an accurate diagnosis of the rheumatic disease due to the scarcity of the preserved bone remains. To perform the DNA extraction of these 90 individuals, dental pieces were preferably selected, since these provide better results than bones when recovering DNA^41,42^. Only in the cases where no dental pieces were preserved, compact bones were selected, preferentially ribs, as this is the anatomic region with the lowest anthropological interest.

## Methods

The diagnosis of rheumatic diseases carried out in the 163 individuals recovered from the site of San Miguel de Ereñozar was described in Laza *et al*.^43^, following the protocol proposed by Ventades *et al*.^44^. The process of DNA extraction was conducted according to the procedure described in Hervella *et al*.^45,46^ and Laza *et al*.^47^. Mitochondrial DNA D-loop hypervariable segment I was sequenced from the aDNA extracts by amplifying six overlapping fragments, each with an approximate length of 100 bp, which finally provided the sequencing between nucleotides 15,995 and 16,399^48^. In the cases in which individuals presented the revised Cambridge Reference Sequence (rCRS) haplotype, a fragment (7F/7R) of mtDNA D-loop Hypervariable Segment II was sequenced, which contains position 73^45,46^. In those individuals who carried polymorphism A073G, PCR-RFLPs were conducted with *Alu*I restriction enzyme to determine the nucleotide in position 7025, with the aim of verifying whether this sequence corresponded to haplogroup H. The mtDNA sequences obtained were aligned with the BioEdit software and then filtered using the databases Haplogrep (https://haplogrep.uibk.ac.at) and Phylotree (https://www.phylotree.org), in order to determine the haplogroup, sub-haplogroup and mitochondrial haplotype of the analysed samples^49,50^.

The following were some of the authentication criteria applied in this study: (1) controls of DNA extraction and amplification (extraction blank and negative control of the PCR), (2) fluorimetric quantification (QUBIT) of the number of template DNA molecules recovered from the extracts obtained, validating the reproducibility of the results, and (3) replication of the results by independent researchers at different times^51–53^.

For the data analysis, several principal component analyses (PCAs) were conducted using the SPSS Statistics v24 software, with the aim of interpreting the covariation of the bone pathologies and mitochondrial lineages in the analysed individuals. The Fst distance and the genetic diversity of different groups were calculated taking into account the frequencies of mtDNA haplogroups and sub-haplogroups using Arlequin 3.5.2.2.

## Results

The analysis of mtDNA in 90 adult individuals of the Ereño necropolis allowed identifying 7 different mitochondrial haplogroups (H, U, T, K, R, J, HV) (Table 1). The most frequent haplogroup was H (73.3%), which is also the most frequent haplogroup in the current population of Europe (55–40%) and in the region of Busturialdea-Urdaibai, where the Ereño necropolis is located^28,30,31,54–56^. The Ereño necropolis shows higher frequencies than the region of Busturialdea-Urdaibai for haplogroups T and R, and lower frequencies for HV, J and U; the frequencies for haplogroup K are similar in both populations (Table 1)^56^. The differences between the two populations were statistically significant (p_value_ = 0.02).

**Table 1:** Frequency of mitochondrial haplogroups in the medieval necropolis analysed in the present study (Ereño, 13^th^–16^th^ centuries) and in the current population where the Ereño necropolis is located, i.e., the region of Busturialdea-Urdaibai (Bizkaia, Spain)^56^.

Haplogroups	% Ereño necropolis (N = 90)	% Busturialdea-Urdaibai (N = 158)
H	73.3	55.1
U	10.0	15.2
T	6.7	4.4
K	3.3	3.2
R	3.3	—
J	2.2	10.1
HV	1.1	7.0
X	—	3.2
I	—	1.3
W	—	0.6

In the present study, 18 mitochondrial sub-haplogroups were defined in the Ereño necropolis. The most frequent sub-haplogroup is H2 (36.7%), followed by sub-haplogroups H1, H3 and T2 (Table 2); all of them have higher frequencies in the analysed necropolis with respect to the current population of Busturialdea-Urdaibai^56^. However, other sub-haplogroups, such as U5, J1 and HV, show lower frequencies in the studied necropolis than in the current population of Busturialdea-Urdaibai (Table 2)^56^, with significant differences between the two populations ( p_value_ = 0.045).

**Table 2:** Frequency of the mitochondrial sub-haplogroups in the medieval necropolis analysed in the present study (Ereño, 13^th^–16^th^ centuries) and in the current population where the Ereño necropolis is located, i.e., the region of Busturialdea-Urdaibai (Bizkaia, Spain)^56^.

Sub-haplogroups	% Ereño necropolis (N = 90)	% Busturialdea-Urdaibai (N = 158)
H2	36.67	31.65
H1	21.11	16.46
H3	6.67	3.16
T2	6.67	3.80
U5	5.56	12.66
H14	3.33	—
K* (K1, K2)	3.33	3.2
R8	3.33	—
H5	2.22	1.27
J1	2.22	8.86
U*	2.22	—
H7	1.11	—
H11	1.11	—
H24	1.11	—
HV*	1.11	10.6
U1	1.11	—
U3	1.11	—
X*	—	3.2
I*	—	1.3
W*	—	0.6

The analysis of the mitochondrial haplotypes allowed identifying the presence of 55 different haplotypes in the 90 individuals analysed from the medieval necropolis of Ereño, which demonstrates the high variability of the population of this site (Supplementary Table S1).

Regarding the rheumatic pathologies of the 90 adult individuals of the Ereño necropolis, 47 of them (52.2%) had some type of joint pathology and 43 (47.7%) did not have any pathological bone manifestations. Of the 7 mitochondrial haplogroups identified (H, HV, J, K, R, T, U), 5 were found in the individuals with rheumatic pathologies (H, J, R, T, U), whereas all 7 haplogroups were identified in the individuals without bone pathologies, with the absence of haplogroups HV and K in the first group (Supplementary Fig. S1). The individuals with rheumatic pathologies showed a lower diversity of mitochondrial haplogroups (0.3691) compared to the individuals without rheumatic pathologies (0.5360), although no statistically significant differences were found between the two groups ( p_value_ = 0.23).

The results of the mitochondrial haplogroups regarding joint pathologies show that 56.1% of the individuals who carried haplogroup H had joint lesions, whereas the rest of the individuals (43.9%) did not have signs of arthropathy (Supplementary Fig. S1). Although the other 6 haplogroups had a much lower frequency than haplogroup H, it is worth mentioning that, in the case of haplogroup U (10%) (Table 1), it was observed that 66.7% of the individuals had rheumatic lesions, whereas 33.3% showed no joint bone lesions (Supplementary Fig. S1). Regarding haplogroup T (6.7%) (Table 1), lower frequency values were obtained in the individuals with joint bone pathologies (33.3%) (Supplementary Fig. S1). Haplogroups K, R, J and HV had a very low frequency in this population (<5%); in fact, haplogroups K and HV were not found in individuals with rheumatic pathologies (Table 1, Supplementary Fig. S1).

**Figure 1:**
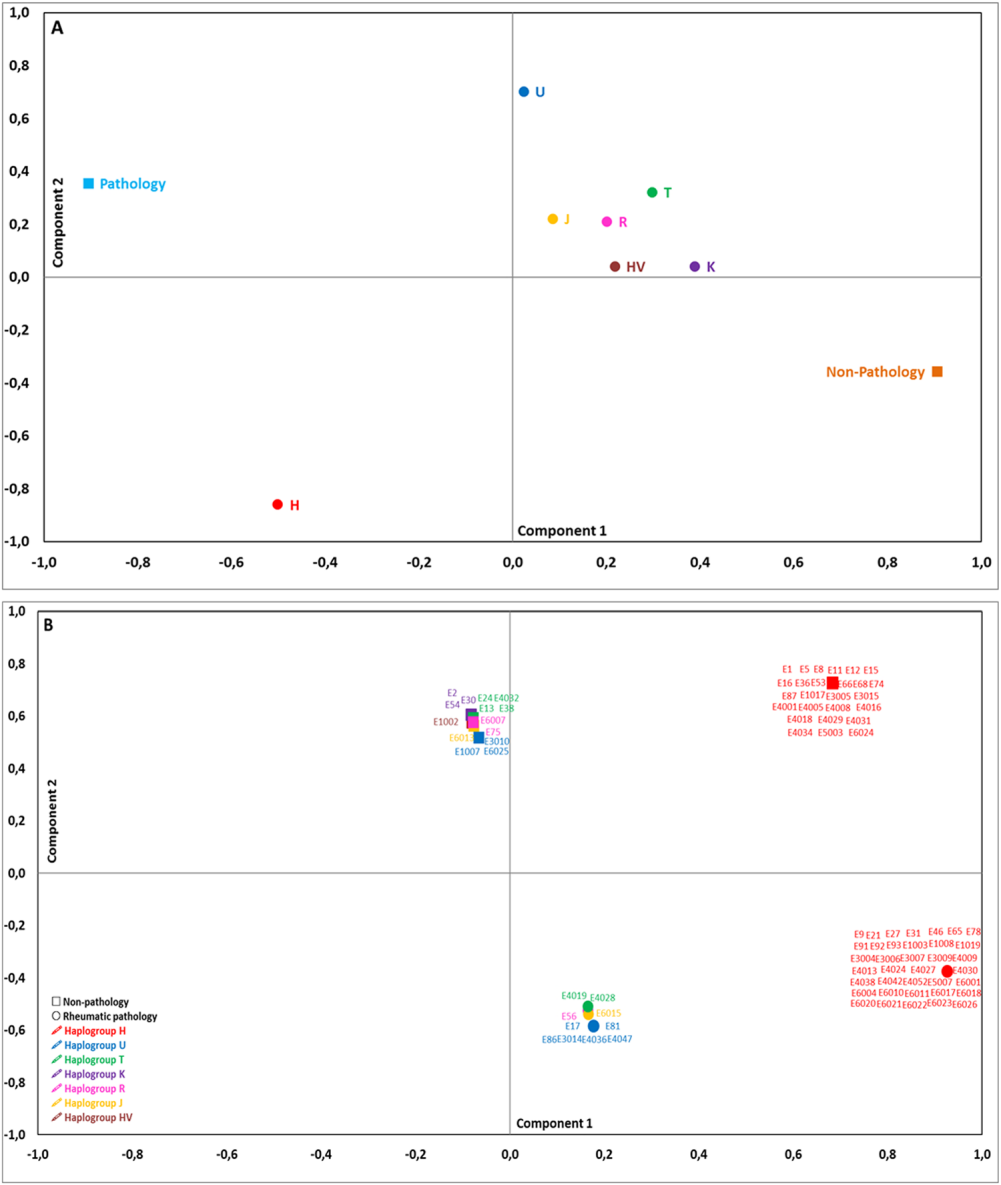
Principal component analysis of the frequency of the mitochondrial haplogroups and the variables pathology and non-pathology of the 90 individuals recovered from the medieval necropolis of San Miguel de Ereñozar (Ereño, Bizkaia, Spain, 13^th^–16^th^ centuries). (**A**) Distribution of the variables pathology and non-pathology and the mitochondrial haplogroups. (**B**) Distribution of the individuals according to the variables pathology and non-pathology and the mitochondrial haplogroups. Component 1: 50.7% of the variance; component 2: 31.5% of the variance.

In order to obtain a more global view of the variability of the individuals of the Ereño necropolis (N = 90), a PCA was conducted considering the mitochondrial haplogroups of both groups, i.e., individuals diagnosed with joint pathologies (N = 47) and without joint pathologies (N = 43), which generated two principal components that explained 82.2% of the variance (Fig. 1A). In PCA axis 1 (50.7% of the variance), the variables with the highest correlation values were: joint pathology (Pathology, −0.906) and absence of joint pathology (Non-Pathology, 0.906). In the second component (31.5% of the variance), the variables with the highest correlation values were: haplogroup H (H, −0.859) and haplogroup U (U, 0.703).

When representing the individuals on the two principal components of the PCA (Fig. 1B), we found that in the most positive end of component 1 there is a group characterised for having rheumatic pathologies and carrying haplogroup H. In the most positive end of the second component of the PCA there are the individuals who had no rheumatic lesions, and in the most negative end there are the individuals that did show joint lesions, regardless of the mitochondrial haplogroup carried by any of these two groups. Considering the 2 axes of the PCA, it is worth highlighting that the most differentiated individuals were those who had rheumatic pathologies and carried haplogroup H, which indicates that haplogroup H would be more related to those individuals who had a joint pathology. Therefore, the next step was to analyse the distribution of the haplogroups in the individuals who had a particular rheumatic disease (spondyloarthropathy, osteoarthritis, rheumatoid arthritis and rheumatic pathology without an accurate diagnosis).

When analysing the mitochondrial variability only in the individuals with rheumatic diseases (N = 47), 5 different haplogroups were identified (H, U, T, R, J), with H being the most frequent one, followed by U; the rest of the haplogroups (T, R, J) showed frequency values below 5%. With respect to the type of rheumatic disease, in the present study it was observed that almost half of the individuals with haplogroup H had spondyloarthropathies (SpA), whereas the other 4 haplogroups represented a small sample size which did not allow any statistical valuation (Supplementary Fig. S2).

**Figure 2:**
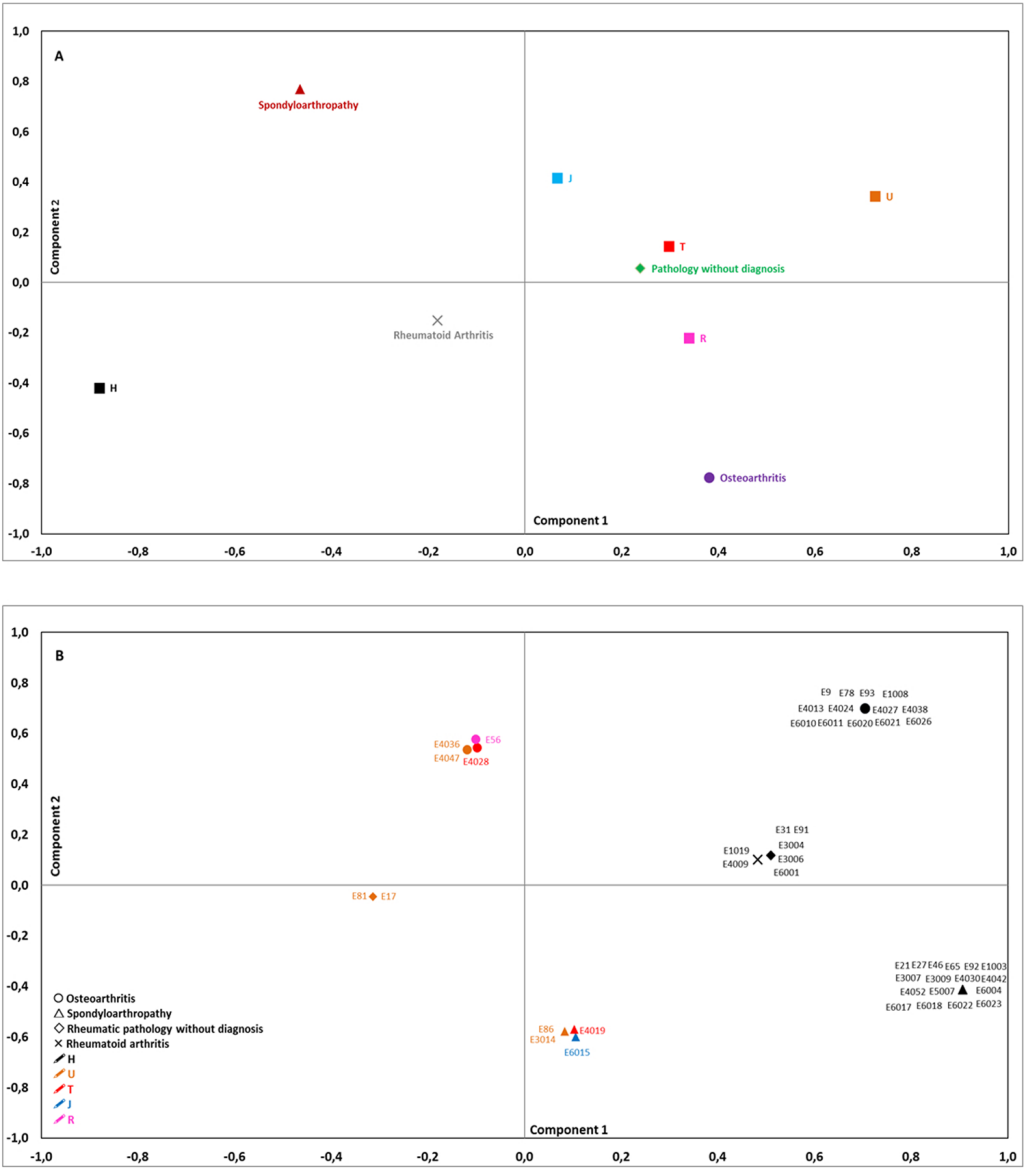
Principal component analysis of the 90 individuals recovered from the medieval necropolis of San Miguel de Ereñozar (Ereño, Bizkaia, 13^th^–16^th^ centuries). (**A**) Distribution of the variables: rheumatic diseases and mitochondrial haplogroups. (**B**) Distribution of the individuals according to the rheumatic diseases and mitochondrial haplogroups they showed in each case. Component 1: 47.8% of the variance; component 2: 25.4% of the variance.

A second PCA was carried out in order to analyse the variability of the mitochondrial haplogroups only in the individuals who had rheumatic diseases, which produced two principal components that explained a variance of 73.2% (Fig. 2). The variables with the highest correlation values in PCA axis 1 (47.8% of the variance) were haplogroup H (H, −0.811) and haplogroup U (U, 0.724), and in PCA axis 2 (25.4% of the variance), the variables with the highest correlation values were osteoarthritis (OA, −0.776) and spondyloarthropathy (SpA, 0.770) (Fig. 2A).

When representing the individuals on the two principal components (Fig. 2B), we found the individuals who had SpA and haplogroup H in the most positive end of axis 1. In axis 2, the individuals with OA were in the most positive end and in the most negative end there were those diagnosed with SpA, with both groups carrying different mitochondrial haplogroups. Considering the PCA axes, it is worth highlighting that the most differentiated individuals were those with SpA and haplogroup H, which indicates the covariance of haplogroup H and SpA.

18 different mitochondrial sub-haplogroups were identified in this study (H2, H1, H3, T2, U5, H14, R8, H5, J1, K1, U*, H7, H11, H24, HV*, K2, U1, U3) (Table 2), 8 of which were shared by individuals with and without rheumatic pathologies (H2, H1, H3, T2, U5, H14, R8, J1) (Supplementary Fig. S3).

The most frequent mitochondrial sub-haplogroup found in this necropolis was H2 (36.7%) (Table 2), in which 66.7% of the individuals showed joint lesions (Supplementary Fig. S3). The rest of the mitochondrial sub-haplogroups showed low frequency values, preventing any statistical valuation (Table 2, Supplementary Fig. S3). When we analysed the distribution of the mitochondrial sub-haplogroups in the different rheumatic diseases, we found that the most frequent sub-haplogroup (H2) appeared in 57% of the individuals with SpA, a very high frequency compared to other rheumatic pathologies (Supplementary Fig. S4).

## Discussion

The genetic heritage of the current European populations is the result of numerous demographic and cultural episodes that took place since the arrival of the anatomically modern *Homo sapiens* to Europe around 45,000 years ago^57^. Regarding the mitochondrial lineages, haplogroup H is the most common and diverse in the current European population (55–40%)^28,30,31,54^, and haplogroup U is the oldest European lineage, with a current frequency of 11%^28,32–34^. Both lineages survived in the glacial refugia of Southwestern Europe during the Last Glacial Maximum (LGM)^29–32^, from where they re-expanded to Northern and Eastern Europe.

Among the causes that would explain the distribution of haplogroup H in the current European population, the climate change that took place during the LGM seems to have played an important role. Moreover, it has been described that the demographic changes that occurred during the Neolithic were a key factor for the diversification of haplogroup H and for the increase of its frequency, becoming the main haplogroup at present in Western Europe (55–40%)^28,30,31,58^. The highest frequency has been reported in the populations of the Northern Iberian Peninsula (Basque Country, Asturias and Galicia), decreasing toward Northern and Eastern Europe, with the exception of Wales, where this haplogroup has a frequency of over 50%^54,55^.

Furthermore, some authors have suggested the existence of a relationship between mitochondrial DNA (mtDNA) and rheumatic diseases, based on the critical role that the mitochondrion plays in cellular function and in survival against oxidative stress^59,60^. In the energy generation process, via oxidative phosphorylation, the mitochondrion produces Reactive Oxygen Species (ROS), whose increase causes damage to lipids, proteins and DNA, promoting mitochondrial dysfunction and inflammation in different pathologies^11,59,61,62^.

Different studies have shown that some mitochondrial haplogroups can increase the risk of developing degenerative joint diseases, whereas other haplogroups constitute a protection factor^19,59,63–67^. Haplogroup H could be related to a higher risk of developing degenerative bone diseases^13–18^, whereas other haplogroups, such as J and T, would be associated to a lower risk of developing osteoarthritis (OA) and psoriatic arthritis^13–15,17,19–21^.

In this context, the population recovered from the medieval necropolis of San Miguel de Ereñozar (Ereño, Bizkaia, Basque Country, Spain, 13^th^–16^th^ centuries), allowed us to evaluate the possible relationship between mtDNA and rheumatic diseases, since this population presents three relevant characteristics: (i) a high frequency of mitochondrial haplogroup H, (ii) a chronology that overlaps with an unfavourable climate period (Little Ice Age, LIA; 14^th^–19^th^ centuries), and (iii) a high prevalence of rheumatic diseases (30%), which were diagnosed as spondyloarthropathies (SpA) (45%), osteoarthritis (OA) (36%) and rheumatoid arthritis (RA) (4%), with a 15% frequency of rheumatic pathologies that could not be accurately diagnosed.

In the present study, we analysed the mitochondrial variability of 90 adult individuals of the medieval necropolis of Ereño, finding 7 haplogroups (H, U, T, K, R, J and HV) and 18 different sub-haplogroups (Tables 1 and 2). The most frequent mitochondrial haplogroup was H (73.3%), whose frequency is higher than that reported in the current population of Busturialdea-Urdaibai (Table 1)^56^. These two populations showed statistical significant differences due to the high frequency of haplogroup H in the necropolis of Ereño.

The analysis of the mitochondrial haplogroups revealed the existence of 55 different haplotypes among the 90 individuals, with haplotype rCRS presenting a very high frequency in the necropolis (≃24%) (Table S1), although similar to the values found in the current populations of the Basque Country (27-21%)^55,56^. Therefore, we can discard the existence of endogamy among the individuals of the necropolis.

When analysing the relationship between haplogroup H and rheumatic diseases, it was observed in the present study that 56% of the individuals that carried this haplogroup had rheumatic lesions, compared to 44% of the individuals who did not show these bone pathologies, which suggests that carrying haplogroup H can pose a greater risk of developing rheumatic diseases (Supplementary Fig. S1). This relationship would be supported by the hypothesis that haplogroup H would be related to a greater oxidative stress, greater cartilage degradation and higher risk of developing degenerative bone pathologies^13–18^. In the present study, it was observed that haplogroup H was more frequent among the individuals who had bone manifestations characteristic of SpA (81%) (Supplementary Fig. S2, Fig. 2B), although this haplogroup is also present with lower frequencies in individuals diagnosed with other rheumatic diseases, such as OA and RA (Supplementary Fig. S2). Regarding the H sub-haplogroups identified in the Ereño necropolis, H2 (the most frequent one, 36.7%), showed differences in the individuals with joint manifestations with respect to those who did not have these manifestations (Supplementary Fig. S3). This result suggests that this sub-haplogroup would be strongly related to a higher risk of developing rheumatic diseases. Moreover, H2 was identified in 57% of the individuals diagnosed with SpA (Supplementary Figure S4), suggesting that sub-haplogroup H2 increases the risk of developing SpA.

The other mitochondrial haplogroups (U, T, K, R, J, HV) showed a small sample size, which does not allow determining their role in the susceptibility of developing rheumatic diseases (Table 1, Supplementary Fig. S1).

The proposed relationships between these mitochondrial haplogroups and their protective or non-protective role in different pathologies are based on the nature of the mitochondrion, in which, by means of the Oxidative Phosphorylation System (OXPHOS), energy is generated to produce heat and thus maintain the body temperature, as well as ROS (Reactive Oxygen Species)^25,27^. Different studies have suggested that European haplogroups would have been molded by selective mechanisms related to low temperatures during the last glacial period, when *Homo sapiens* dispersed over Europe^25–27,68^. In the present study, we propose that the temperature decrease that took place in the Little Ice Age (LIA) was an environmental factor that influenced the selection of some mitochondrial haplogroups over others, favouring those haplogroups that were more efficient at obtaining energy and heat from food to endure lower temperatures and food shortage.

Haplogroup H, one of the most energy-efficient mitochondrial lineages, has a high frequency in the medieval population of this study that lived through the LIA, a period in which energy demands would have risen. However, this haplogroup produces a high oxidative stress, cell damage and cartilage degeneration, promoting the development of degenerative bone diseases^24,27,69,70^. In this study, we observed that the individuals who carried haplogroup H had a significantly high frequency of rheumatic pathologies, more specifically spondyloarthropathies, which was also verified for sub-haplogroup H2.

## Conclusions

From the evolutionary perspective, the high frequency of haplogroup H found in the population of San Miguel de Ereñozar (Basque Country, Spain) could be considered as a result of a biological adaptation related to adverse environmental conditions, such as the ones that took place during the Little Ice Age, since this mitochondrial haplogroup is more energy-efficient at enduring and surviving these conditions. However, this would imply a biological trade-off, increasing the risk of developing rheumatic diseases.

## Supplementary information

Supplementary Information

Supplementary Information 2
