# Supplementary material for: Environmental factors modulated ancient mitochondrial DNA variability and the prevalence of rheumatic diseases in the Basque Country
Source: Sci Rep. 2019 Dec 31;9:20380. doi: 10.1038/s41598-019-56921-x (PMC6938509; doi:10.1038/s41598-019-56921-x)
Supplement: Supplementary file 1 — Supplementary Information [file 41598_2019_56921_MOESM1_ESM.pdf]

Environmental factors modulated ancient mitochondrial DNA variability and the prevalence of rheumatic diseases in the Basque Country

Laza IM[1]*, Hervella M[1], Neira Zubieta M[2], de-la-Rúa C[1]

1 Department of Genetics, Physical Anthropology and Animal Physiology, Faculty of Science and Technology, University of the Basque Country (UPV-EHU); Leioa; Biscay; Basque Country; 48940; Spain.

2 QarK Arqueología. Vitoria-Gasteiz; Álava; Basque Country; 01006; Spain.

*Correspondence to imanol.martinl@ehu.eus

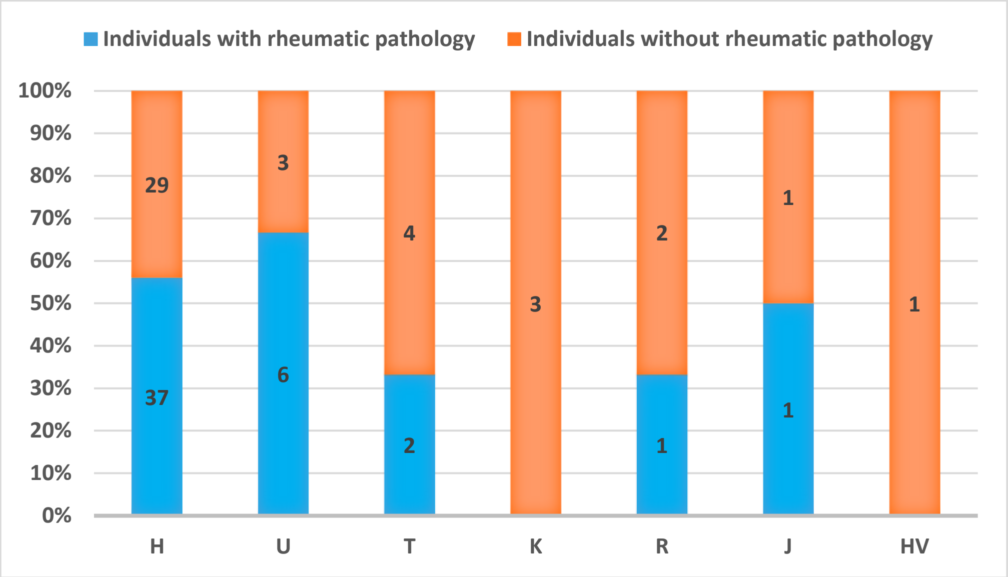

Supplementary Figure S1. Distribution of the frequency of the mitochondrial haplogroups in the medieval population of San Miguel de Ereñozar (N = 90).

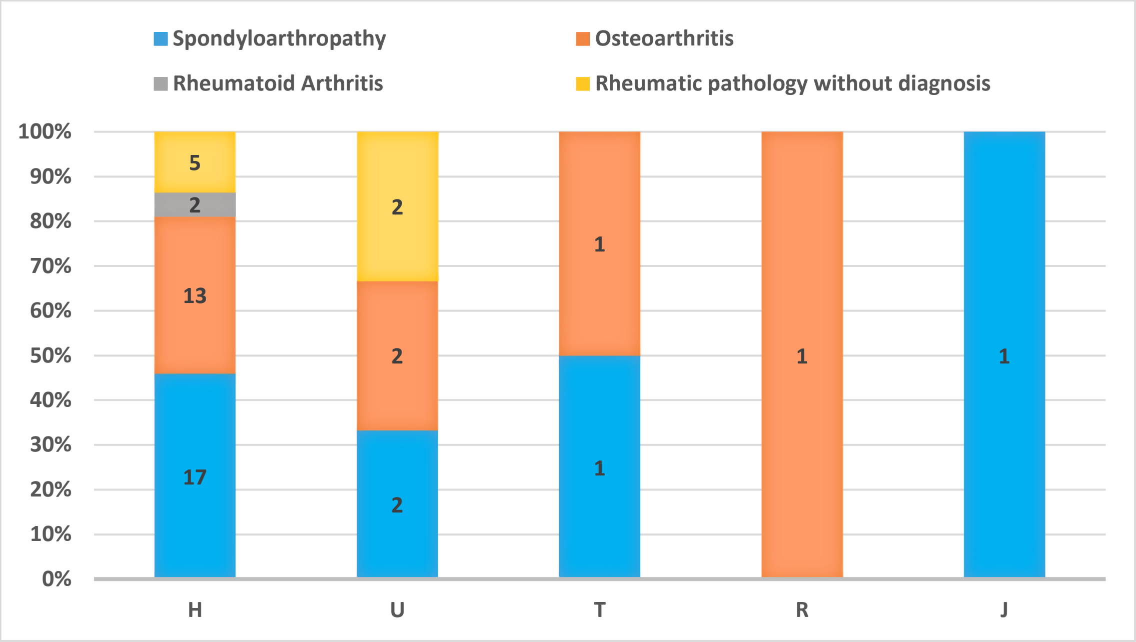

Supplementary Figure S2. Distribution of the frequencies of the mitochondrial haplogroups in the analysed individuals with rheumatic pathologies from the medieval necropolis of San Miguel de Ereñozar. The frequency of the haplogroups is itemised according to the type of rheumatic pathology.

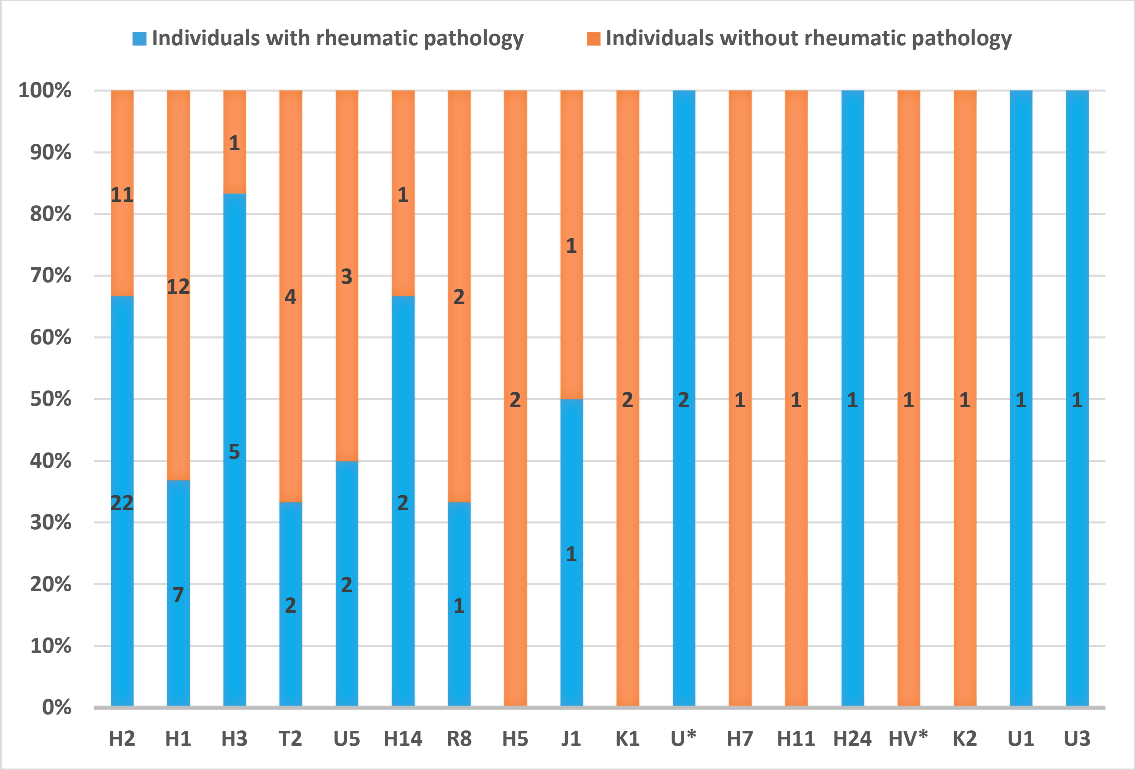

Supplementary Figure S3. Distribution of the frequencies of the mitochondrial sub-haplogroups identified in the individuals with rheumatic pathologies (N = 47) and without rheumatic pathologies (N = 43) recovered from the medieval necropolis of San Miguel de Ereñozar (N = 90).

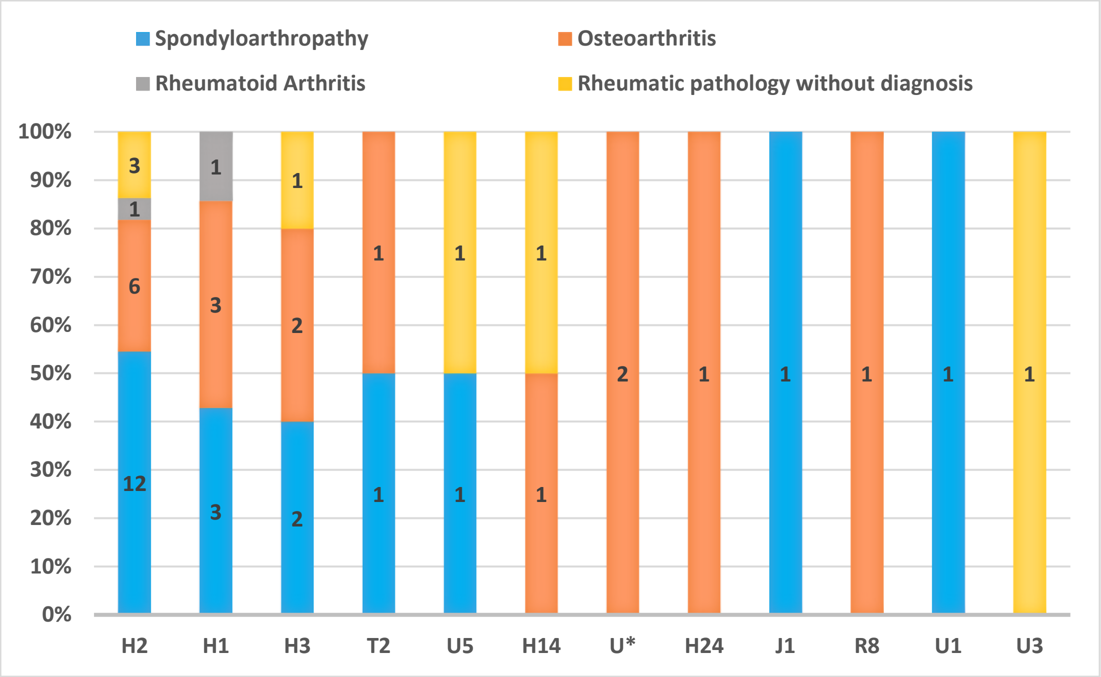

Supplementary Figure S4. Distribution of the frequencies of the mitochondrial sub-haplogroups according to the type of rheumatic pathology in the medieval necropolis of San Miguel de Ereñozar.
